# Exposure to Inorganic Mercury Causes Oxidative Stress, Cell Death, and Functional Deficits in the Motor Cortex

**DOI:** 10.3389/fnmol.2018.00125

**Published:** 2018-05-15

**Authors:** Francisco B. Teixeira, Ana C. A. de Oliveira, Luana K. R. Leão, Nathália C. F. Fagundes, Rafael M. Fernandes, Luanna M. P. Fernandes, Márcia C. F. da Silva, Lilian L. Amado, Fernanda E. S. Sagica, Edivaldo H. C. de Oliveira, Maria E. Crespo-Lopez, Cristiane S. F. Maia, Rafael R. Lima

**Affiliations:** ^1^Laboratory of Functional and Structural Biology, Institute of Biological Sciences, Federal University of Pará, Belém, Brazil; ^2^Laboratory of Ecotoxicology, Institute of Biological Sciences, Federal University of Pará, Belém, Brazil; ^3^Laboratório de Cultura de Tecidos e Citogenética, SAMAM, Instituto Evandro Chagas, Ananindeua, Brazil; ^4^Laboratory of Molecular Pharmacology, Institute of Biological Sciences, Federal University of Pará, Belém, Brazil; ^5^Laboratory of Inflammation and Behavior Pharmacology, Pharmacy Faculty, Institute of Health Science, Federal University of Pará, Belém, Brazil

**Keywords:** mercury, mercury chloride, cell death, oxidative stress, motor cortex

## Abstract

Mercury is a toxic metal that can be found in the environment in three different forms – elemental, organic and inorganic. Inorganic mercury has a lower liposolubility, which results in a lower organism absorption and reduced passage through the blood–brain barrier. For this reason, exposure models that use inorganic mercury in rats in order to evaluate its effects on the central nervous system are rare, especially in adult subjects. This study investigated if a chronic exposure to low doses of mercury chloride (HgCl2), an inorganic form of mercury, is capable of promoting motor alterations and neurodegenerative in the motor cortex of adult rats. Forty animals were exposed to a dose of 0.375 mg/kg/day, for 45 days. They were then submitted to motor evaluation and euthanized to collect the motor cortex. Measurement of mercury deposited in the brain parenchyma, evaluation of oxidative balance, quantification of cellular cytotoxicity and apoptosis and density of mature neurons and astrocytes of the motor cortex were performed. It was observed that chronic exposure to inorganic mercury caused a decrease in balance and fine motor coordination, formation of mercury deposits and oxidative stress verified by the increase of lipoperoxidation and nitrite concentration and a decrease of the total antioxidant capacity. In addition, we found that this model of exposure to inorganic mercury caused cell death by cytotoxicity and induction of apoptosis with a decreased number of neurons and astrocytes in the motor cortex. Our results provide evidence that exposure to inorganic mercury in low doses, even in spite of its poor ability to cross biological barriers, is still capable of inducing motor deficits, cell death by cytotoxicity and apoptosis, and oxidative stress in the motor cortex of adult rats.

## Introduction

Mercury is the third most toxic element on the planet, according to the US Government Agency for Toxic Substances and Disease Registry (Rice et al., 2014). This substance can be found in three different forms in the environment: (i) elemental mercury or metallic mercury (Hg^0^); (ii) inorganic mercury (i.e., mercuric chloride, HgCl_2_); and (iii) organic mercury (i.e., methylmercury, MeHg) (Bernhoft, 2012).

Mercury compounds are bioaccumulative and toxic pollutants, and their anthropogenic natural emissions represent a high risk to human health, thus becoming a global concern (Giang and Selin, 2016; Wang et al., 2016). Mercury caused public health disasters such as those that occurred in Minamata Bay in Japan (Ministry of the Environment from Japan, 2013) and Iraq (Skerfvingi and Copplestone, 1976; Clarkson et al., 1981).

The toxic properties, biological behavior, toxicokinetics and clinical manifestations of mercury compounds are directly related to their chemical forms (Bernhoft, 2012; Rice et al., 2014). Studies involving the effects of inorganic mercury in adults are barely seen in the literature, due to factors such as low liposolubility, low corporal absorption and low passage through the blood–brain barrier (BBB) (Bernhoft, 2012; Rice et al., 2014).

Throughout the years, inorganic mercury has been used in drugs, dermatologic lotions and germicide solutions, exposing human beings to their toxic effects. In fact, anthropogenic activity (i.e., artisanal gold mining) has exposed humans to chronic intoxication by inorganic mercury, which leads to deleterious health effects (Doering et al., 2016). The literature on intoxication and inorganic mercury exposure in humans consists of reports of clinical cases (Triunfante et al., 2009; Benz et al., 2011; Beasley et al., 2014).

Inorganic mercury has been reported to damage the kidney (Peixoto and Pereira, 2007; de Freitas et al., 2012; Gado and Aldahmash, 2013; Joshi et al., 2014), liver (Trebucobich et al., 2014), gastrointestinal tract (Bernhoft, 2012), cardiovascular system (Da Cunha et al., 2000; Golpon et al., 2003; Omanwar et al., 2011, 2013) and reproductive system (El-Desoky et al., 2013; Kalender et al., 2013). In addition, it has already been shown that this mercurial species crosses the placental barrier and causes changes in the fetus (Heath et al., 2009; Chehimi et al., 2012). In these latter works, neurotoxicity is highlighted as a deleterious consequence of intoxication with inorganic mercury.

Most studies on the neurotoxicity of inorganic mercury are carried out with experimental models of different cerebral development stages (gestational, pre-natal or postnatal period) (Szász et al., 2002; Huang et al., 2011; Chehimi et al., 2012), due to the immaturity of the BBB. In contrast, studies in adult organisms are scarce (Mello-Carpes et al., 2013; Teixeira et al., 2014a).

In our previous study (Teixeira et al., 2014a), we reported that inorganic mercury is able to accumulate in the brain parenchyma and that this finding is associated with functional alterations. Consequently, we evaluated the possible changes that chronic exposure to inorganic mercury in low doses causes at the behavioral, tissue and biochemical level to assess the possible damage this multilevel model can reveal.

## Materials and Methods

### Ethics Statement

The animal protocols used in this work were evaluated and approved by the Ethics Committee on Experimental Animals of the Federal University of Pará (Protocol BIO139-13). They are in accordance with the NIH Guide for the Care and Use of Laboratory Animals and national law for laboratory experimentation (National Research Council of the National Academies, 2011).

### Animals and Experimental Groups

**FIGURE 1:**
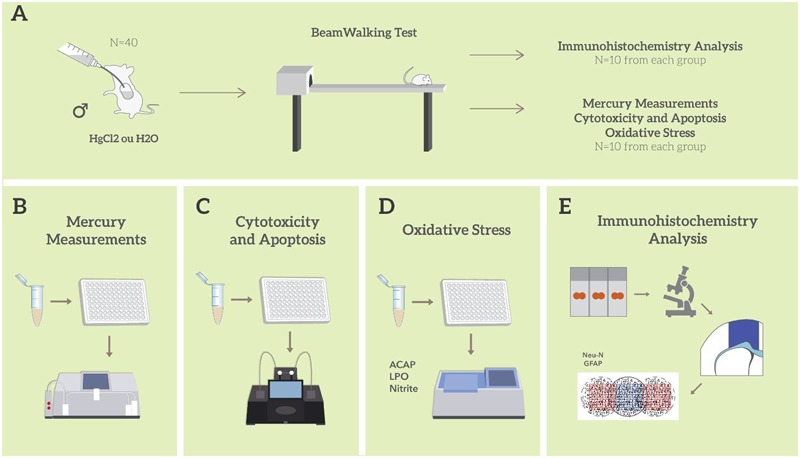
Sample description and experimental stages. Description of the sample and model of exposure to HgCl_2_; Beam walking motor test; division of experimental groups and animal destinations for each stage of analysis **(A)**; total mercury measurement assay **(B)**; evaluation and quantification of cytotoxicity and apoptosis **(C)**; oxidative balance assays **(D)**; immunohistochemistry and morphometric analysis **(E)**. All tissue and biochemical assays were performed in triplicate.

Male Wistar rats (*n* = 40; 90 days old) were obtained from the Federal University of Pará (UFPA) and kept in collective cages (five animals per cage). Animals were maintained in a climate-controlled room on a 12-h reverse light/dark cycle (lights on 7:00 a.m.), with food and water *ad libitum*. Distilled water or mercury chloride (HgCl_2_) (dose of 0.375 mg/kg/day) were administered orally (gavage) over a period of 45 days (i.e., until the 135th day of life), according to a procedure previously described by Teixeira et al. (2014a). The animals were weighed weekly for dose adjustment. The experimental design is summarized in **Figure 1**.

### Behavioral Assay

#### Beam Walking Test

Motor coordination and balance were assessed using the beam walking test. The apparatus consists of a wooden beam (100 cm) suspended 50 cm from the floor, which allows the animal to access a secure platform (closed box of 20 × 20 cm). Initially, the rats were acclimated in the beams of higher cross-sectional area, and a limit of 120 s was fixed to reach the box. In order to intensify the task difficulty, animals were submitted to two test sessions (cut-off 60 s each) on the square (28, 12, and 5 mm diameter) and round (28, 17, and 11 mm diameter) beams, respectively (Carter et al., 1999). The latency to reach the closed box and the number of foot slips were measured in seconds (one or both hind limbs slipped from the beam) (Karl et al., 2003).

After the end of behavioral test, the animals were assigned to different analyses. Ten animals from each group were perfused and destined for morphological brain analysis. The other 10 were sacrificed by cervical displacement, their brains immediately removed, the motor cortex dissected and destined for biochemical and neurochemical evaluations.

### Mercury Measurements

For mercury measurement, each homogenized sample of motor cortex was weighted (0.5 *g* maximum of wet weight) in a sample digestion bottle, and 1 mL of distilled water, 2 mL of nitric acid-perchloric acid with HNO_3_-HClO_4_ (equal proportions, 1 + 1) and 5 mL of sulfuric acid (H_2_SO_4_) were sequentially added, followed by heat treatment on a hot plate (200–230°C) for 30 min. The final volume (50 ml) was completed by distilled water. The extracts transferred to 0 and 1.0 mL of methylmercury-cysteine solution in two sample digestion bottles and (corresponding to 0 and 0.10 μg.Hg) and 1 mL of distilled water was added only to the former (the blank) followed by 2 mL of HNO_3_-HClO_4_ and 5 mL of H_2_SO_4_. In order to obtain blank and standard test solutions for the measurement of total mercury, the same procedure for the sample test solution was followed. Total mercury content in the samples was estimated by wet digestion, reduction and cold vapor atomic absorption spectrometry (Semi-automated Mercury Analyzer, model Hg-201, Sanso Seisakusho Co. Ltd., Tokyo, Japan); the circulation-open airflow system was as previously described by Suzuki et al. (2004). Mercury measurements were calculated by the following formula (Akagi and Nishimura, 1991): total mercury concentration in the sample (μg/g) = 0.10 μg × (test sample – blank sample)/(standard sample – blank sample) × dilution factor × 1/sample weight (g) × ratio of wet weight/dry weight.

All analyses were conducted in triplicates of the group tissue samples and the values obtained ranged from a confidence interval of ±10% (*r*: 0.9992).

### Oxidative Balance Analysis

#### Sample Preparation

The motor cortex samples collected for biochemical analysis were frozen in liquid nitrogen and stored at -80°C. They were then thawed and resuspended in Tris-HCl 20 mM, pH 7.4 at 4°C and sonically disaggregated (∼1 g/mL). To avoid extensive damage to proteins and lipids, we carry out the homogenization in ice-cold buffer for a few seconds each time and treat all the samples in exactly the same way. The lysate was stored at -80°C until processing time. All biochemical assays were performed in triplicate.

#### Antioxidant Capacity Against Peroxyl Radicals (ACAP)

Total antioxidant competence against peroxyl radicals was analyzed through ROS determination in samples incubated or not with a peroxyl radical generator. The detailed methodology is described in Lowry et al. (1951) and Amado et al. (2009). Peroxyl radicals were produced by thermal (35°C) decomposition of 2, 2′-azobis 2 methylpropionamidine dihydrochloride (ABAP; 4 mM; Aldrich). For ROS determination we employed the fluorogenic compound 2′,7′-dichlorofluorescin diacetate (H2DCF-DA) at a final concentration of 40 mM. H2DCF-DA passively diffuses through cellular membranes and once inside the acetates are cleaved by intracellular esterases. Thereafter, the non-fluorescent compound H2DCF is oxidized by ROS to the fluorescent compound DCF. The readings were performed in a fluorescence microplate reader (Victor 2, Perkin Elmer) every 5 min for 1 h. Background fluorescence was determined before the addition of DCF-DA. Total fluorescence production was calculated by integrating the fluorescence units (FU; *y* axis) along the time of the measurement (*x* axis), after adjusting FU data to a second order polynomial function. The results were calculated as area difference of FU × min in the same sample with and without ABAP addition and standardized to the ROS area without ABAP (background area). Using this methodology, a reduced relative area means higher antioxidant capacity, because low fluorescence levels obtained after the addition of ABAP, indicate high competence in neutralizing peroxyl radicals. For a direct reading of the results, the inverse of relative difference between ROS area with and without ABAP was considered a measure of antioxidant capacity.

#### Quantification of Nitrite

An aliquot of crude homogenate was centrifuged at 21,000 *g* for 20 min at 4°C, and the supernatant was used to analyze nitrite levels as described in Green et al. (1982). Briefly, the samples were incubated at room temperature for 20 min with Griess reagent (0.1% naphthyl-ethylene diamine and 1% sulfanilamide in 5% phosphoric acid – 1: 1). The absorbance was measured at 550 nm in a spectrophotometer and compared to the standard sodium nitrite solutions.

#### Lipid Peroxidation Assay

Lipid peroxidation was estimated as the levels of malondialdehyde (MDA) and 4-hydroxyalkenes (4HDA) as detailed earlier by Esterbauer and Cheeseman (1990). Briefly, an aliquot of crude homogenate was centrifuged at 2500 *g* for 30 min at 4°C, and 200 μL of the supernatant was incubated with 650 μL of *N*-methyl-2-phenylindole 10.3 mM and methanol (1:3). After mixed, 150 μL of methanosulfonic acid was added and incubated at 45°C for 40 min. The absorbance was measured at 570 nm and compared to those of standard solutions of MDA.

#### Protein Concentration Assay

The measurement of the amount of proteins in the supernatants (used for the determination of lipid peroxidation, nitrite levels and ACAP) was performed as described by Lowry et al. (1951). Thus, after correction for the protein concentration, the results of lipid peroxidation, nitrites and ACAP were expressed as percentages of the mean of the control group.

### Evaluation and Quantification of Cytotoxicity and Apoptosis

In these evaluations, motor cortex samples were treated with collagenase at concentrations of 2 mg/ml and 4 mg/mL, respectively, for tissue dissociation and subsequently stored at 37°C for 20 min and 40 min. Thereafter, 100 μL of solution containing the isolated cells was added into 96 well microplates with 100 μL of the Cytotox-glo^TM^ Cytotoxicity Assay, which uses a luminogenic peptide substrate to measure dead-cell protease activity or Caspase-Glo^®^ 3/7 Assay Systems, a luminescent assay to measure caspase-3/7 activities (Promega Systems). Readings were performed on the GloMax^®^ (Promega, Netherlands) according to the manufacturer’s recommendations. The quantification results were expressed as relative fluorescence units (RFU) for cytotoxicity or relative luminescence units (RLU) for apoptosis, both relative to the control group.

### Histological Evaluation

#### Perfusion and Histological Procedures

After the motor test, part of the animals (*n* = 10 animals per group) were deeply anesthetized with ketamine hydrochloride (90 mg/kg, i.p.) and xylazine hydrochloride (10 mg/kg, i.p.) and submitted to transcardiac perfusion with an heparinized saline 0.9% solution followed by 4% paraformaldehyde in 0.2 M phosphate buffer. Surgical manipulation was performed only after the removal of the corneal and paw reflexes. The brains were removed from the skull and postfixed for 6 h in Bouin’s solution. After this period, the specimens were washed with 50% alcohol, dehydrated in alcohol solutions in progressive concentrations clarified in xylol and embedded in paraffin.

#### Immunohistochemistry

Coronal brain sections (5 μm thick) were submitted to immunohistochemical analysis. Immunohistochemistry procedures have been described in our previous investigations (Teixeira et al., 2014b; Oliveira et al., 2015). The samples were sectioned and placed on silanized slides. The slides were dewaxed in xylol and hydrated in ethanol at increasing concentrations. Antigenic recovery was performed with citrate buffer pH 6.0, followed by endogenous peroxidase blocking in 3% hydrogen peroxide solution in methanol for 20 min. Sections were then washed in a 0.05% Tween PBS solution for 5 min, three times and incubated with 10% normal horse serum in PBS (anti-NeuN) and goat serum (anti-GFAP) for 1 h. Sections were then incubated overnight with the primary antibodies diluted in PBS, anti-NeuN (1: 500, Milipore, United States) and anti-GFAP (1: 1000, Sigma, United States). Afterward, sections were washed in PBS/Tween solution for 5 min (three times) and incubated with biotinylated horse anti-mouse (anti-NeuN) and goat anti-rabbit (GFAP) secondary antibodies diluted 1:500 in PBS for 2 h. Sections were washed again for five min (three times) and incubated with the avidin-biotin peroxidase complex (ABC kit, Vector Laboratories, United States) for 2 h. Finally, the sections were washed four times with PBS/Tween (5 min each) and developed with Diaminobenzidine (DAB). After the reaction, the sections were washed twice in PB 0.1M, dehydrated and covered with coverslips with Entellan (Merck, Germany).

#### Morphometric Analysis

All sections were initially evaluated by light microscopy. Illustrative images of all experimental groups were obtained with a digital camera coupled to a microscope. Coronal sections containing the motor cortex were used to count the number of neurons (NeuN + cells) and astrocytes (GFAP + cells) using a square gradient of 0.0665 mm^2^ coupled to the optical microscope eyepiece (40x). Four fields per section and two sections per animal were counted for the control and experimental groups. The detailed counting methodology is described in previously published studies (Oliveira et al., 2014; Teixeira et al., 2014b; Fontes-Júnior et al., 2016).

### Statistical Analysis

All data (*n* = 20 animals per group for behavioral tests and *n* = 10 per group for other analyses) were statistically analyzed for their normality using the Shapiro–Wilk test. Statistical comparison of body weight was performed using one-way analysis of variance (ANOVA) with repeated measures (days) followed by a Tukey’s *post hoc* test. For comparative statistics between groups, Student’s *t*-test was used. The Pearson correlation test was performed for the oxidative balance data in the group exposed to HgCl_2_. The value of *p* ≤ 0.05 was considered statistically significant. GraphPad Prism 5.0 software (San Diego, CA, United States) was used for statistical analysis.

## Results

**FIGURE 2:**
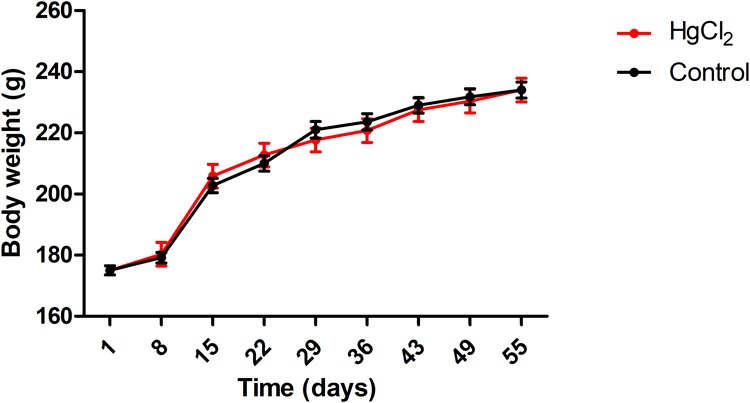
Effects of chronic exposure to HgCl_2_ on body weight of adult Wistar rats (g). Results are expressed as mean ± standard error after the one-way ANOVA for repeated measures analysis. Standard error and a one-way ANOVA analysis revealed that there were no significant differences (*p* > 0.05) between groups in any time.

**FIGURE 3:**
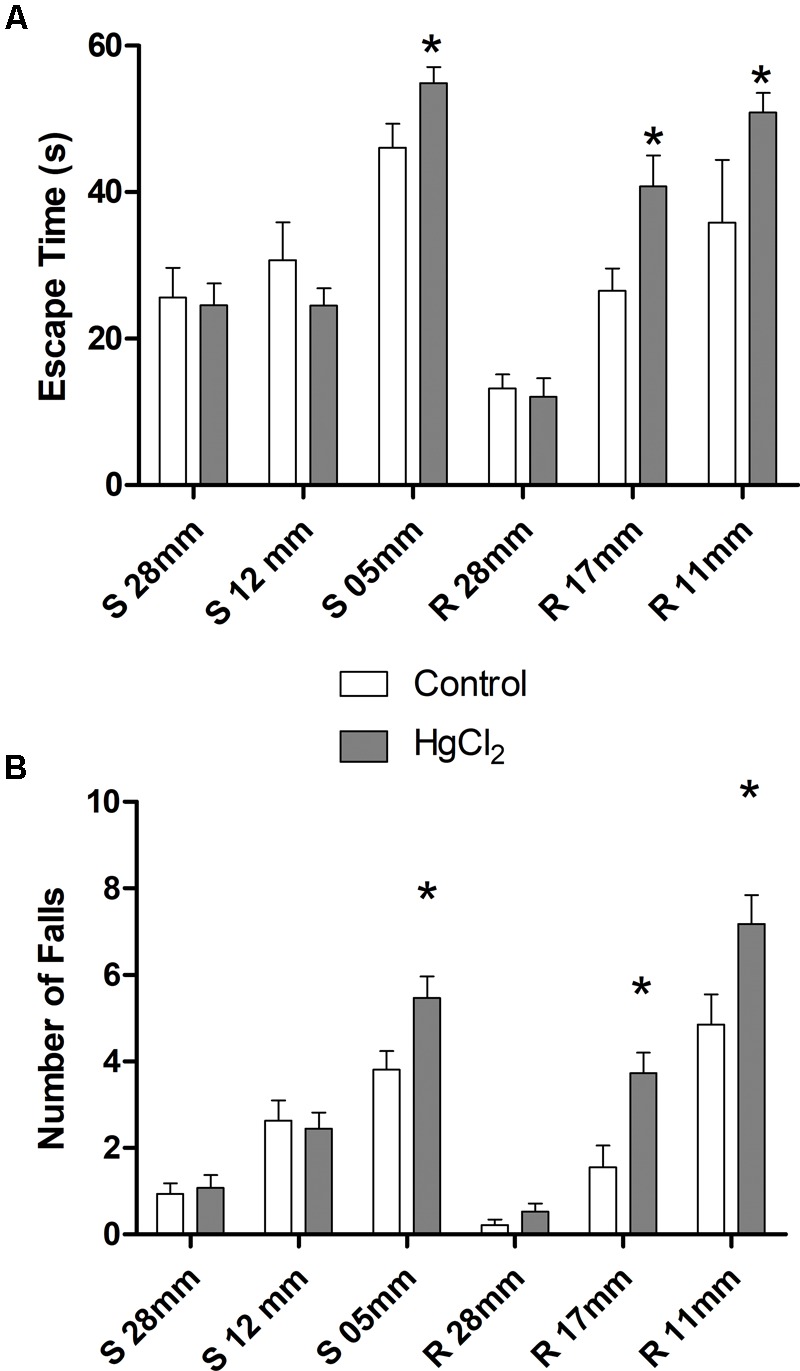
Effects of chronic exposure to HgCl_2_ on fine motor coordination and balance of adult Wistar rats. The results are expressed as mean ± standard error of **(A)** latency (s) and **(B)** failure numbers. ^∗^*p* < 0.05 compared to the control group (Student’s *t*-test).

**FIGURE 4:**
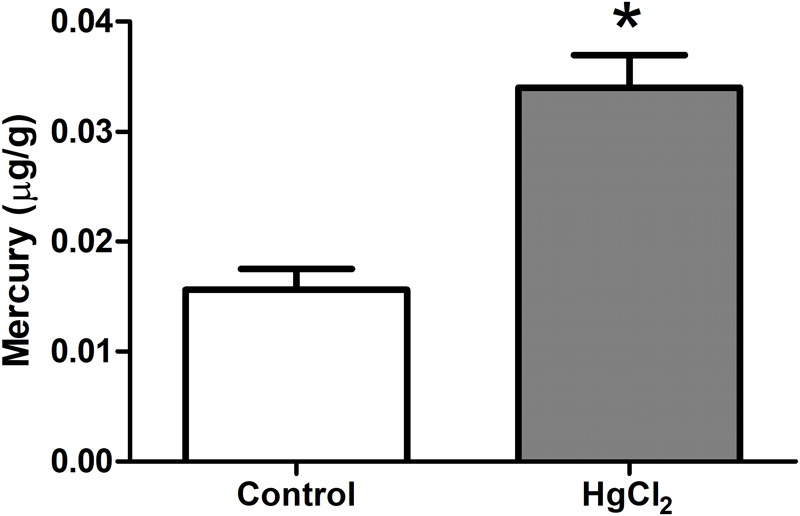
Effects of chronic exposure to HgCl_2_ on the formation of mercury deposits in the brain parenchyma in the motor cortex of adult Wistar rats. Results are expressed as mean ± standard error. ^∗^*p* < 0.05 compared to the control group (Student’s *t*-test).

One-way ANOVA with repeated measures followed by a Tukey’s *post hoc* test revealed that chronic exposure to HgCl_2_ did not alter the body weight of the animals during all protocol (*P* > 0.05 for all values; **Figure 2**). No deaths were observed during the exposure period. The experimental group exposed to the inorganic mercury presented a decrease in the latency and an increase in the number of failures during the challenges with the smaller square (5 mm: latency *p* = 0.0351, number of failures *p* = 0.0388) and round beams (17 mm: latency *p* = 0.0328, number of failures *p* = 0.0061; and 11 mm: latency *p* = 0.0418, number of failures *p* = 0.0388) proposed by the beam walking test. The results are shown in **Figure 3**. Moreover, the exposure for 45 days to inorganic mercury increased the mercury concentration deposited in the parenchyma of the motor cortex (**Figure 4**).

**FIGURE 5:**
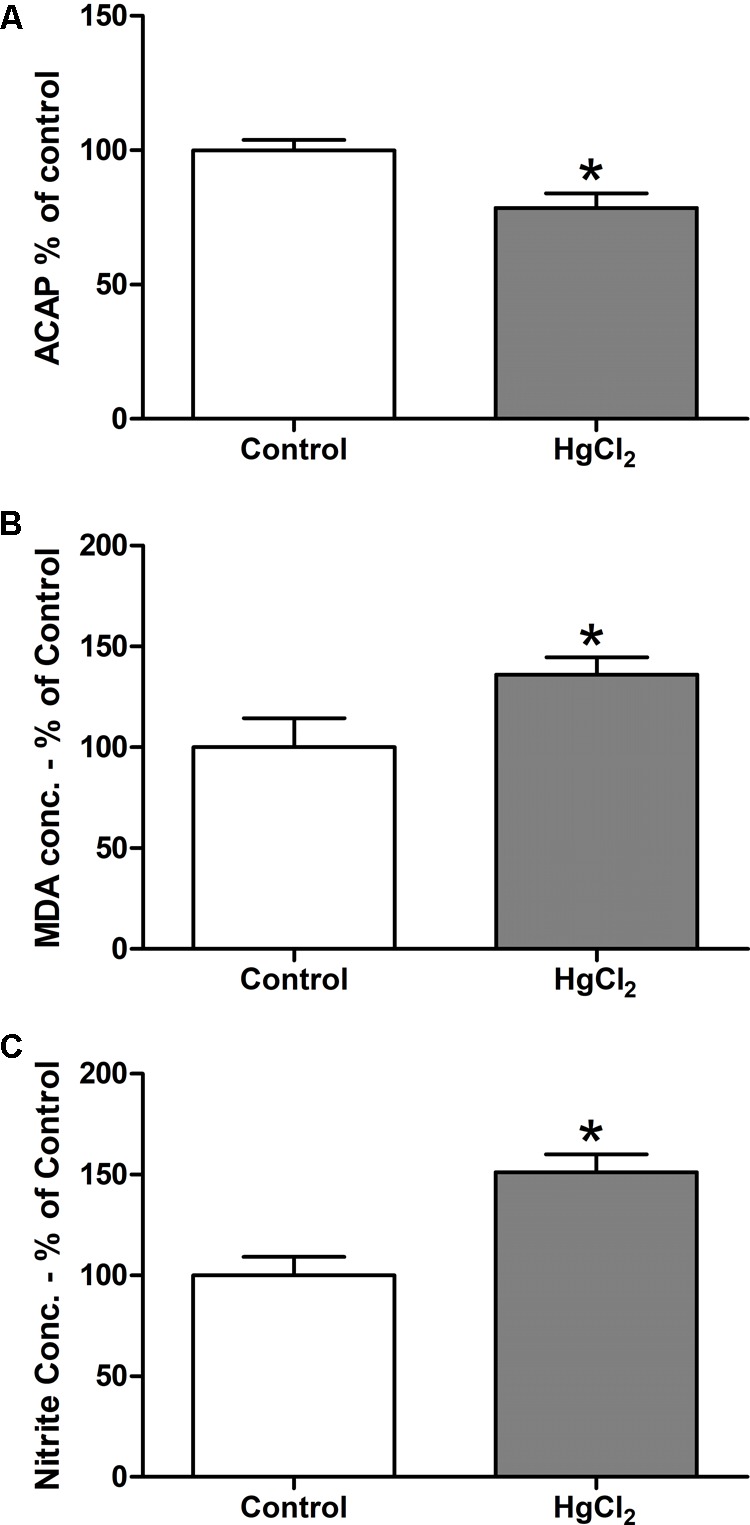
Effects of chronic exposure to HgCl_2_ on oxidative balance in the motor cortex of adult Wistar rats. The results are expressed as mean ± standard error of the **(A)** percentages of the fluorescence unit area difference of the generated curves of the same sample with and without ABAP in comparison to the control group; **(B)** percentage of malondialdehyde over control in mg, normalyzed per mg of protein; and **(C)** percentage of nitrite over control in mg, normalyzed per mg of protein. ^∗^*p* < 0.05 compared to the control group (Student’s *t*-test).

**FIGURE 6:**
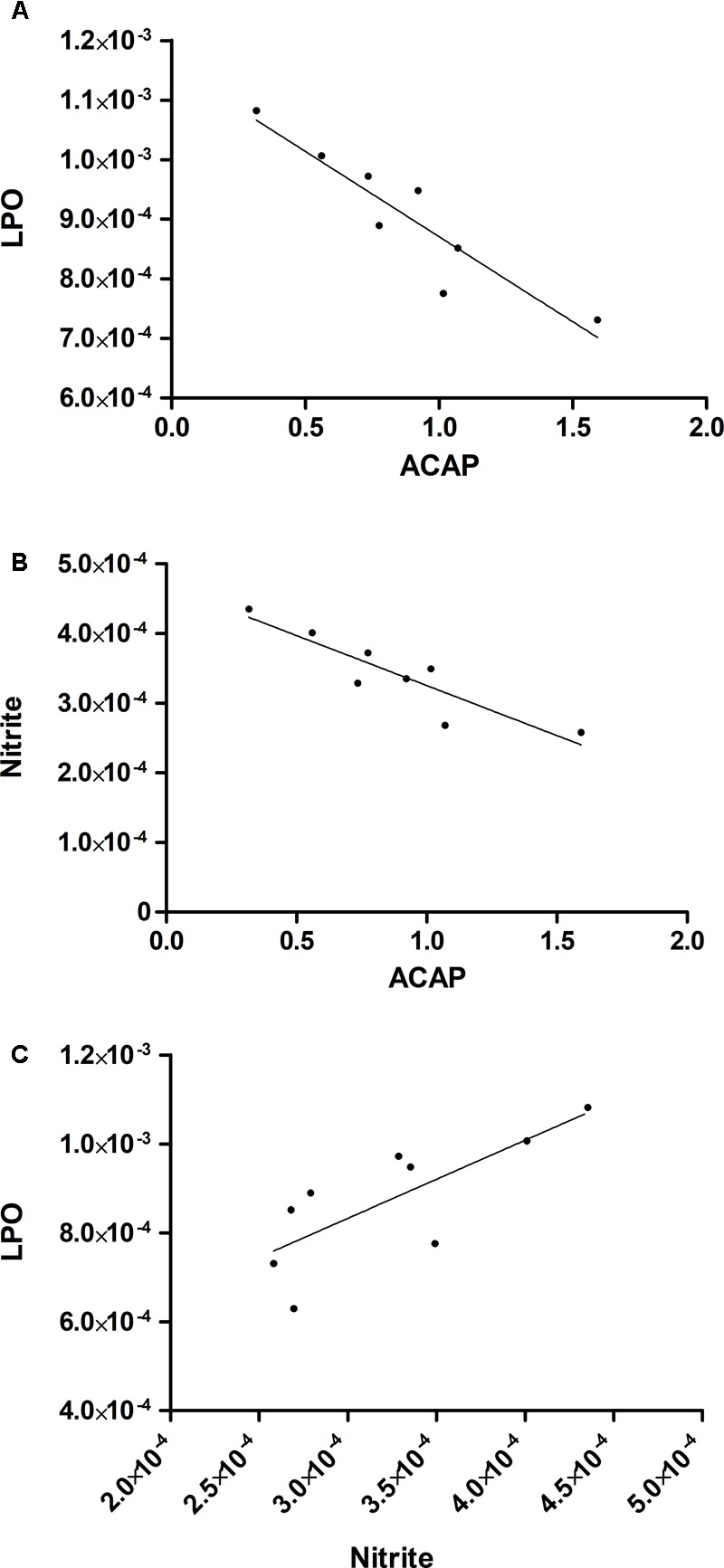
The increase in levels of pro-oxidant measures is associated with a decrease in total antioxidant capacity. The results are expressed by the intersection between the correlated data. **(A)** Negative correlation between ACAP and malondialdehyde levels; **(B)** negative correlation between ACAP and nitrite levels; and **(C)** positive correlation between malondialdehyde and nitrite levels. The analysis was performed by Pearson’s correlation.

The tested model of exposure to inorganic mercury decreased the antioxidant capacity in the motor cortex (*p* = 0.0367) (**Figure 5A**). Moreover, the chronic exposure to HgCl_2_ increased the levels of pro-oxidant parameters in the motor cortex of rats, as demonstrated by increased levels of malondialdehyde – a lipid peroxidation indicator – (*p* = 0.0396) (**Figure 5B**) and of nitrite levels – an indirect marker of nitrite oxide production (*p* = 0.0014) (**Figure 5C**). A negative correlation, classified as robust, was observed between MDA levels and ACAP (*r* = -0.91; *p* = 0.0012) (**Figure 6A**). A negative correlation was also identified between nitrite levels and ACAP (*r* = -0.73; *p* = 0.01) (**Figure 6B**). A positive and strong correlation was observed between MDA levels and nitrite levels (*r* = 0.76; *p* = 0.01) (**Figure 6C**).

**FIGURE 7:**
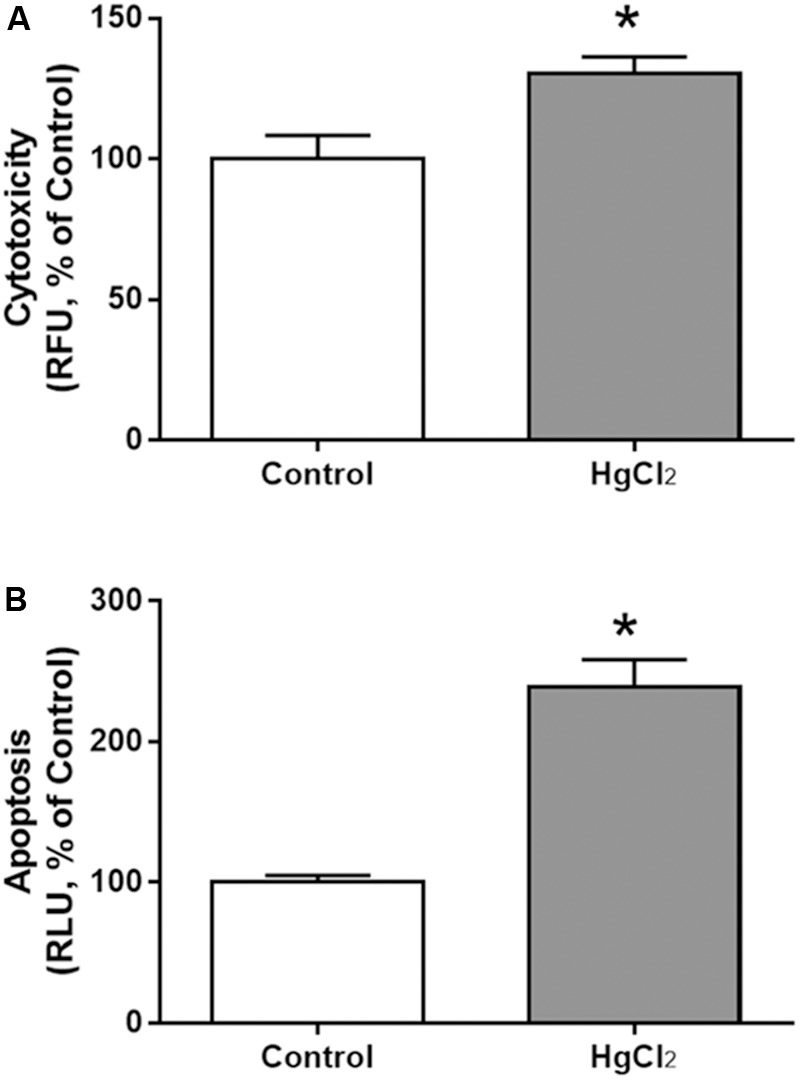
Effects of chronic exposure to HgCl_2_ on cytotoxicity and induction of apoptosis in the motor cortex of adult Wistar rats. The results are expressed as mean ± standard error of the **(A)** percentages of cytotoxicity at relative fluorescence units (RFU) relative to the control group and **(B)** percentages of apoptosis at relative luminescence units (RLU) in relation to the control group. ^∗^*p* < 0.05 compared to the control group (Student’s *t*-test).

**FIGURE 8:**
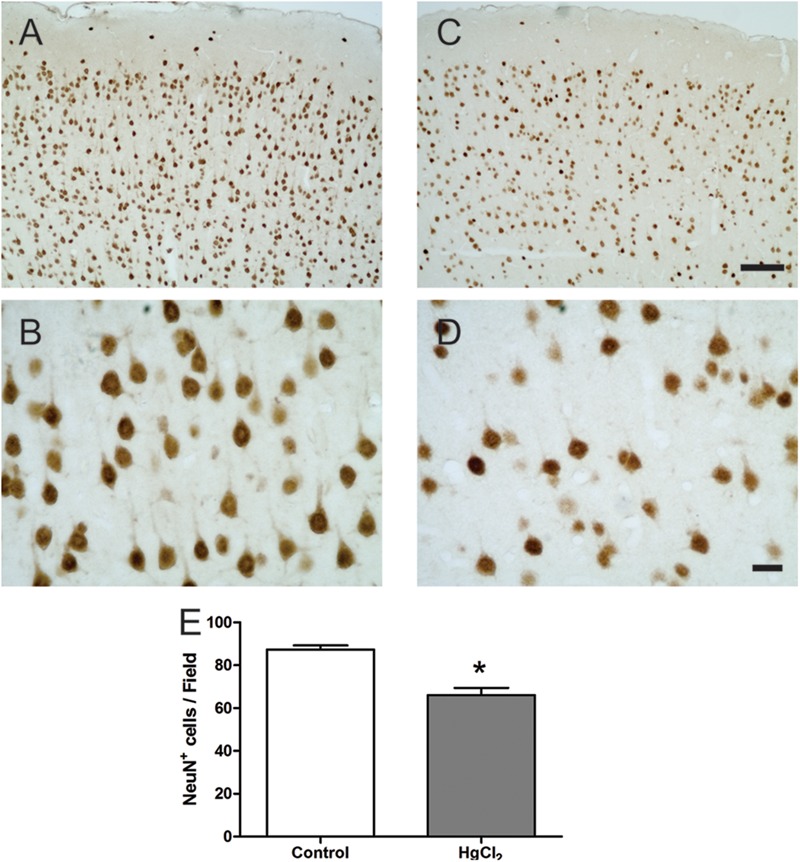
Effects of chronic exposure to HgCl_2_ on neuronal density in the motor cortex of adult Wistar rats. **(A–D)** represent photomicrographs of the NeuN-labeled motor cortex of the control animals **(A,B)** and the exposed animals **(C,D)**. The results are expressed as mean ± standard error of the number of cells counted per field **(E)**. ^∗^*p* < 0.05 compared to the control group (Student’s *t*-test). Scale bar **(A,C)** 100 μm; **(B,D)** 20 μm.

**FIGURE 9:**
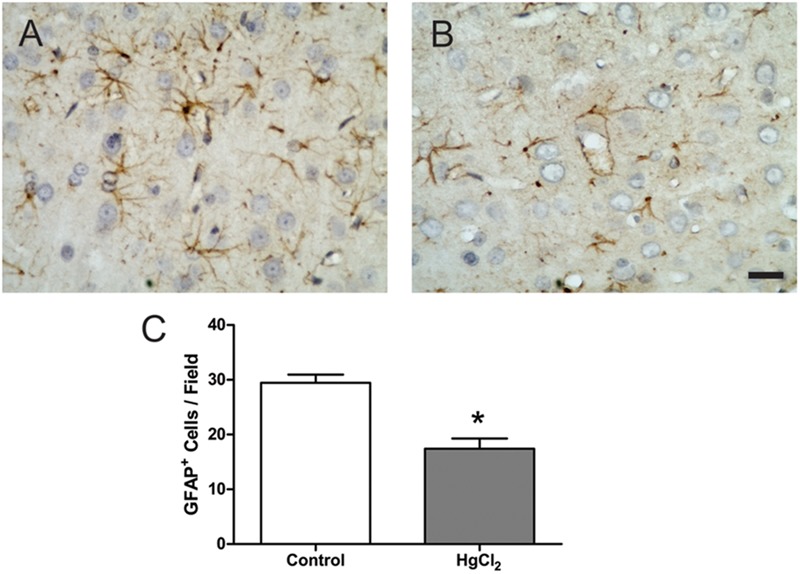
Effects of chronic exposure to HgCl_2_ on astrocyte density in the motor cortex of adult Wistar rats. **(A,B)** Represent photomicrographs of the GFAP-labeled motor cortex of control animals **(A)** and exposed animals **(B)**. Results are expressed as mean ± standard error of the number of cells counted per field **(C)**. ^∗^*p* < 0.05 compared to the control group (Student’s *t*-test). Scale bar 20 μm.

Chronic exposure to HgCl_2_ induced cytotoxicity (*p* = 0.0294) (**Figure 7A**) and apoptosis (*p* = 0.0077) (**Figure 7B**) in the motor cortex. These results indicate that inorganic mercury causes cell death in the central nervous system. Moreover, the immunohistochemical identification of mature neurons (NeuN) (**Figure 8**) and astrocytes (GFAP) (**Figure 9**) indicated that chronic exposure to HgCl_2_ decreased the immunoreactivity of NeuN (*p* = 0.0159) (**Figure 8E**) and GFAP + (*p* = 0.0286) (**Figure 9C**).

## Discussion

This study shows, for the first time, that chronic exposure to low doses of inorganic mercury in adult rats leads to mercury deposits in the brain parenchyma associated with oxidative stress and cell death by cytotoxicity and induction of apoptosis, which deteriorates motor function.

Models of chronic exposure to inorganic mercury are relatively uncommon in the literature, although the oral route was already showed to be most important during chronic exposure to inorganic mercury (see Introduction). Unfortunately, scarce information is available about inorganic mercury burden in chronically exposed populations because epidemiological studies usually analyzed the total mercury content and did not analyze the levels of the different mercury species (Berzas-Nevado et al., 2010). These reasons probably prompted Szász et al. (2002) to develop their interesting model linking the average daily water consumption of the rats during mercury treatments to the tolerance of the treated animals to different levels of mercury poisoning (e.g., birth rate, developmental disturbances, mortality). These authors used 0.8 mg HgCl2/kg body weight per day (3.99 mmol/kg per day), which were tolerated both by the pregnant dams, as well as by their pups without obvious sign of toxicity (no movement disorder for adults and average body weight of newborns no less than 85% of controls).

In our work, we used less than a half of that dose, characterizing an exposure to relatively low levels of inorganic mercury. However, this exposure was enough to cause significant mercury deposits in the motor cortex (**Figure 4**). Although the low liposolubility of inorganic mercury may prevent it from directly crossing the BBB (Bernhoft, 2012; Rice et al., 2014), it was already demonstrated that the biological barriers do not avoid the deleterious effects of this compound in brain and fetus (Szász et al., 2002; Huang et al., 2011; Chehimi et al., 2012).

Although mechanistic studies about the pharmacokinetics of inorganic mercury are scarce and additional studies are necessary, one hypothesis to explain how inorganic mercury can reach the CNS is because of the altered integrity of the BBB. Exposure to this compound causes alterations of the transport enzymes (such as Na^+^/K^+^ ATPase) of cortical microvessels and perivascular swelling (Szumañska et al., 1993).

Moreover, inorganic mercury appears to have a particular tendency to accumulate in the cortex rather than in other brain regions such as the hippocampus (Teixeira et al., 2014a), significantly altering gross motor coordination in addition to memory (Teixeira et al., 2014a). Additional behavioral assays such as the beam walking test are more informative about motor refinement, motor coordination and balance (Carter et al., 1999; Karl et al., 2003). This motor test allows a detailed evaluation of subtle motor impairments, due to the gradual increase of the challenge imposed on the animal in crossing narrow beams (Carter et al., 1999; Karl et al., 2003). In our work, higher mercury deposits in the motor cortex were associated with to disability in tasks requiring greater motor refinement, as shown by the poor performance of exposed animals on the smaller diameter beams (square 5 cm and circular of 11 and 17 mm).

Although alterations in balance and fine motor coordination are usually associated to cerebellar damage (Karl et al., 2003), the motor cortex plays an important role in fine motor control and fractionation of movement, sensorimotor integration and higher order cognitive–motor movements (Asanuma, 1973; Evarts et al., 1983; Sanes and Donoghue, 2000). Moreover, chronic exposure in animals seems to cause similar accumulations of inorganic mercury in cerebellum and temporal and frontal lobes (Ostertag et al., 2013). Therefore, according to our results (**Figures 3, 4**), although other brain areas (including the cerebellum and basal ganglia) could be also participating in the deleterious consequences of the exposure, the motor cortex may plays a major role in the motor deficiencies caused by inorganic mercury.

Our results demonstrated that behavioral alterations were associated with both cellular and molecular damage (**Figures 5–9**). In the motor cortex, cellular densities of astrocytes and neurons were decreased after inorganic mercury exposure (**Figures 8, 9**). Previous toxicological studies already indicate that the decrease in the number of both astrocytes and neurons is usually associated with functional damage observed in behavioral tests (Akagi and Nishimura, 1991; Suzuki et al., 2004; Flores-Montoya et al., 2015; Maodaa et al., 2016). In addition, *in vitro* exposure to inorganic mercury causes cell death in human neurons and astrocytes (Lohren et al., 2015). The latter study proposed that neurons may be more sensitive than astrocytes to the acute intoxication with inorganic mercury. Interestingly, we demonstrated that chronic exposure to relative low levels of inorganic mercury proportionally decreased cortical astrocytes population more than neuronal density (**Figures 8, 9**). Although additional studies are necessary, in this case it is possible that a metal accumulation in glial cells takes place, higher than that of cells of neuronal origin, as it was already demonstrated for other species of mercury such as methylmercury (Berzas Nevado et al., 2009). The long-term effect of this higher accumulation in astrocytes would result on a higher mortality index for cells of glial origin. To our knowledge, this the first time that a different response of astrocytic and neuronal populations to inorganic mercury exposure is demonstrated *in vivo.*

This decrease in cortical cellular populations was associated with induction of apoptosis and cytotoxicity (**Figure 7**). Interestingly, acute exposure to inorganic mercury did not induce a significant apoptosis, as revealed by caspase-3 activity in cells of both neuronal and glial origin (Lohren et al., 2015). However, our results demonstrated that a chronic exposure to the metal is able to cause more than twice the apoptosis of non-exposed animals (**Figure 7**). Moreover, considering that the luminescence method used in our work is based on caspases-3 and 7 activities, our results already suggest different responses according to the type of exposure (chronic or acute). Additionally, a special sensitivity of the cortical tissue cannot be ruled out since this was an *in vivo* study.

A significant cytotoxicity of the cortex was also detected (**Figure 7**). The molecular mechanisms underlying IHg cytotoxicity may include the disruption of lysosomal and cell membrane integrity, the increase of extracellular glutamate levels and the decrease of both mitochondrial activity and intracellular ATP levels, as already demonstrated for the acute *in vitro* exposure to IHg (Lohren et al., 2015; Moneim, 2015). Mainly, the increase of extracellular glutamate levels (caused by both increased release and decreased uptake) would lead to a pronounced overproduction of free radicals such as nitric oxide. Increased levels of free radicals (especially nitric oxide) are found in both brain and serum of animals exposed to inorganic mercury (Gutierrez et al., 2006; Moneim, 2015). Accordingly, our results show increased levels of nitrites (an indirect marker of nitric oxide production) in exposed animals (**Figures 5, 6**). Moreover, this is the first time that this was demonstrated in cortical tissue, being additionally associated with behavioral alterations and cellular death.

Endogenous antioxidant molecules (mainly glutathione) and enzymes (such as superoxide dismutase, glutathione peroxidase and catalase) are the main defenses of the cell against free radicals. Although previous studies showed glutathione depletion with IHg intoxication, results of antioxidant enzymes are contradictory with both increase and decrease of enzymatic activities after IHg exposure (Gutierrez et al., 2006; Ansar, 2015; Moneim, 2015). Therefore, we decided to evaluate the total antioxidant capacity of the cortex as a more reliable marker than each element alone. In our work, we found that, concomitant with increased levels of nitrites, inorganic mercury caused a significant decrease in total antioxidant capacity of the cortex (**Figures 5, 6**). These two factors, overproduction of free radicals and reduced antioxidant defenses, cause a imbalance that contributes synergistically to damage the macromolecules (DNA, proteins and lipids).

Brain tissue is especially sensitive to the deleterious consequences of this imbalance in the redox status. The brain contains large amounts of polyunsaturated fatty acids present in the membranes that are particularly susceptible to free radical attack and thus, to lipid peroxidation (Lucena et al., 2007; Ansar, 2015). Considering that lipid peroxidation plays a major role in necrosis and cellular death, the exacerbated lipid peroxidation detected in cortex (**Figures 5, 6**) could explain the significant death of both neurons and astrocytes caused by IHg (**Figures 8, 9**).

## Conclusion

**FIGURE 10:**
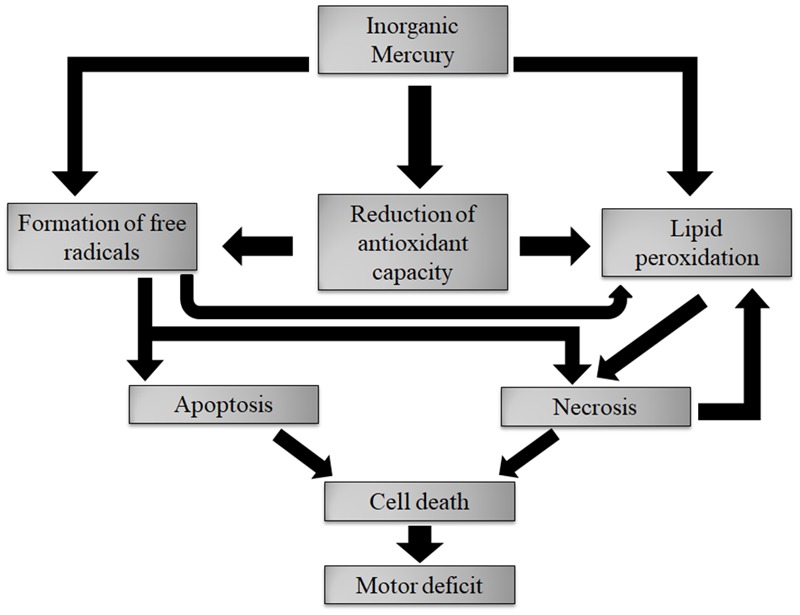
Description of the main results found in this article. Deposits of inorganic mercury caused oxidative stress. The necrosis and apoptosis processes kill cells, both neurons and glia. The cell death is the main factor responsible for fine motor control changes.

After reaching the motor cortex, inorganic mercury accumulated in deposits in this region. This accumulation caused the overproduction of nitric oxide (and maybe other ROS) affecting membrane lipids and causing lipid peroxidation. The necrosis and apoptosis processes killed cells, both neurons and glia. Finally, the decrease in the number of cells was probably the main factor responsible for fine motor control changes – the main findings of this paper are summarized in **Figure 10**.

One of the main contributions of the present work was to show for the first time the consequences of chronic IHg exposure at three different levels simultaneously: clinical (behavioral effects), cellular (cellular death and apoptosis) and molecular (oxidative stress markers). Additionally, a pharmacokinetic marker (mercury content) was evaluated to confirm the exposure. Therefore, we demonstrated that chronic exposure to relatively low levels of inorganic mercury during adulthood causes fine motor disorders associated to cellular death, apoptosis and imbalance of redox status in the cortex. These data rule out the idea that inorganic mercury is innocuous and make clear that it can be quite toxic to particular brain areas even in adults. Complete studies including different levels of analysis are necessary for a better understanding and prevention of IHg intoxication.

## Author Contributions

FT, AO, and RL conceived the study, participated in the experimental design, and wrote the manuscript. LL, NF, LA, and MC-L performed the biochemical analyses. RF performed the immunohistochemistry analyses. CM and LF performed and contributed to analyze cognitive test. MS contributed to measurements Mercury levels. EO and FS performed the citotoxicity and apoptosis assays. All authors read and approved the final manuscript.

## Conflict of Interest Statement

The authors declare that the research was conducted in the absence of any commercial or financial relationships that could be construed as a potential conflict of interest.

## References

[B1] Akagi H. Nishimura H. (1991). “Speciation of mercury in the environment,” in eds Suzuki T. Imura N. Clarkson T. W. (Boston, MA: Springer) 53–76. 10.1007/978-1-4757-9071-9_3

[B2] Amado L. L. Garcia M. L. Ramos P. B. Freitas R. F. Zafalon B. Ferreira J. L. (2009). A method to measure total antioxidant capacity against peroxyl radicals in aquatic organisms: application to evaluate microcystins toxicity. 407 2115–2123. 10.1016/j.scitotenv.2008.11.038 19095287

[B3] Ansar S. (2015). Pretreatment with diallylsulphide modulates mercury-induced neurotoxicity in male rats. 62 599–603. 10.18388/abp.2015_1064 26351819

[B4] Asanuma H. (1973). Cerebral cortical control of movement. 16 143–166. 4197405

[B5] Beasley D. M. G. Schep L. J. Slaughter R. J. Temple W. A. Michell J. M. (2014). Full recovery from a potentially lethal dose of mercuric chloride. 10 40–44. 10.1007/s13181-013-0311-1 23760886 PMC3951629

[B6] Benz M. R. Lee S. H. Kellner L. Döhlemann C. Berweck S. (2011). Hyperintense lesions in brain MRI after exposure to a mercuric chloride-containing skin whitening cream. 170 747–750. 10.1007/s00431-010-1333-1 21052738

[B7] Bernhoft R. A. (2012). Mercury toxicity and treatment: a review of the literature. 2012:46050862. 10.1155/2012/460508 22235210 PMC3253456

[B8] Berzas Nevado J. J. Martín-Doimeadios R. C. R. Jiménez Moreno M. Nascimento J. L. M. Herculano A. M. Crespo-López M. E. (2009). Mercury speciation analysis on cell lines of the human central nervous system to explain genotoxic effects. 93 12–16. 10.1016/j.microc.2009.03.008

[B9] Berzas-Nevado J. J. Rodríguez Martín-Doimeadios R. C. Guzmán Bernardo F. J. Jiménez Moreno M. Herculano A. M. do Nascimento J. L. M. (2010). Mercury in the Tapajós River basin, Brazilian Amazon: a review. 36 593–608. 10.1016/j.envint.2010.03.011 20483161

[B10] Carter R. J. Lione L. A. Humby T. Mangiarini L. Mahal A. Bates G. P. (1999). Characterization of progressive motor deficits in mice transgenic for the human Huntington’s disease mutation. 19 3248–3257. 10.1523/JNEUROSCI.19-08-03248.1999 PMC6782264 10191337

[B11] Chehimi L. Roy V. Jeljeli M. Sakly M. (2012). Chronic exposure to mercuric chloride during gestation affects sensorimotor development and later behaviour in rats. 234 43–50. 10.1016/j.bbr.2012.06.005 22705860

[B12] Clarkson T. W. Magos L. Cox C. Greenwood M. A. Amin-Zaki L. Majeed M. A. (1981). Tests of efficacy of antidotes for removal of methylmercury in human poisoning during the Iraq outbreak. 218 74–83. 7241391

[B13] Da Cunha V. Souza H. P. Rossoni L. V. França A. S. Vassallo D. V. (2000). Effects of mercury on the isolated perfused rat tail vascular bed are endothelium- dependent. 39 124–130. 10790511 10.1007/s002440010001

[B14] de Freitas M. L. da Silva A. R. Roman S. S. Brandão R. (2012). Effects of 4,4′- dichloro-diphenyl diselenide (ClPhSe)2 on toxicity induced by mercuric chloride in mice: a comparative study with diphenyl diselenide (PhSe)2. 34 985–994. 10.1016/j.etap.2012.07.007 22981437

[B15] Doering S. Bose-O’Reilly S. Berger U. (2016). Essential indicators identifying chronic inorganic mercury intoxication: pooled analysis across multiple cross-sectional studies. 11:e0160323. 10.1371/journal.pone.0160323 27575533 PMC5004870

[B16] El-Desoky G. E. Bashandy S. A. Alhazza I. M. Al-Othman Z. A. Aboul-Soud M. A. Yusuf K. (2013). Improvement of mercuric chloride-induced testis injuries and sperm quality deteriorations by *Spirulina platensis* in rats. 8:e59177. 10.1371/journal.pone.0059177 23555627 PMC3610915

[B17] Esterbauer H. Cheeseman K. H. (1990). Determination of aldehydic lipid peroxidation products: malonaldehyde and 4- hydroxynonenal. 186 407–421. 10.1016/0076-6879(90)86134-H 2233308

[B18] Evarts E. V. Fromm C. Kroller J. Jennings V. A. (1983). Motor cortex control of finely graded forces. 49 1199–1215. 10.1152/jn.1983.49.5.1199 6864246

[B19] Flores-Montoya M. G. Alvarez J. M. Sobin C. (2015). Olfactory recognition memory is disrupted in young mice with chronic low-level lead exposure. 236 69–74. 10.1016/j.toxlet.2015.04.013 25936521 PMC4433589

[B20] Fontes-Júnior E. A. Maia C. S. Fernandes L. M. Gomes-Leal W. Costa-Malaquias A. Lima R. R. (2016). Chronic alcohol intoxication and cortical ischemia: study of their comorbidity and the protective effects of minocycline. 2016:1341453. 10.1155/2016/1341453 27418952 PMC4933869

[B21] Gado A. M. Aldahmash B. A. (2013). Antioxidant effect of Arabic gum against mercuric chloride-induced nephrotoxicity. 7 1245–1252. 10.2147/DDDT.S50928 24174869 PMC3808154

[B22] Giang A. Selin N. E. (2016). Benefits of mercury controls for the United States. 113 286–291. 10.1073/pnas.1514395113 26712021 PMC4720344

[B23] Golpon H. A. Puchner A. Barth P. Welte T. Wichert P. V. Feddersen C. O. (2003). Nitric oxide-dependent vasorelaxation and endothelial cell damage caused by mercury chloride. 192 179–188. 10.1016/S0300-483X(03)00303-2 14580785

[B24] Green L. C. Wagner D. A. Glogowski J. Skipper P. L. Wishnok J. S. Tannenbaum S. R. (1982). Analysis of nitrate, nitrite, and [15N] nitrate in biological fluids. 126 131–138. 10.1016/0003-2697(82)90118-X 7181105

[B25] Gutierrez L. L. Mazzotti N. G. Araújo A. S. Klipel R. B. Fernandes T. R. Llesuy S. F. (2006). Peripheral markers of oxidative stress in chronic mercuric chloride intoxication. 39 767–772. 10.1590/S0100-879X2006000600009 16751982

[B26] Heath J. C. Abdelmageed Y. Braden T. D. Nichols A. C. Steffy D. A. (2009). The effects of chronic mercuric chloride ingestion in female Sprague-Dawley rats on fertility and reproduction. 47 1600–1605. 10.1016/j.fct.2009.04.007 19371768 PMC2817961

[B27] Huang C. F. Liu S. H. Hsu C. J. Lin-Shiau S. Y. (2011). Neurotoxicological effects of low-dose methylmercury and mercuric chloride in developing offspring mice. 201 196–204. 10.1016/j.toxlet.2010.12.016 21195143

[B28] Joshi D. Mittal D. K. Shukla S. ASrivastav K. Srivastav S. K. (2014). N-acetyl cysteine and selenium protects mercuric chloride-induced oxidative stress and antioxidant defense system in liver and kidney of rats: a histopathological approach. 28 218–226. 10.1016/j.jtemb.2013.12.006 24485406

[B29] Kalender S. Uzun F. G. Demir F. Uzunhisarcıkli M. Aslanturk A. (2013). Mercuric chloride-induced testicular toxicity in rats and the protective role of sodium selenite and vitamin E. 55 456–462. 10.1016/j.fct.2013.01.024 23369933

[B30] Karl T. Pabst R. von Hörsten S. (2003). Behavioral phenotyping of mice in pharmacological and toxicological research. 55 69–83. 10.1078/0940-2993-00301 12940631

[B31] Lohren H. Blagojevic L. Fitkau R. Ebert F. Schildknecht S. Leist M. (2015). Toxicity of organic and inorganic mercury species in differentiated human neurons and human astrocytes. 32 200–208. 10.1016/j.jtemb.2015.06.008 26302930

[B32] Lowry O. H. Rosebrough N. J. Farr A. L. Randall R. J. (1951). Protein measurement with the folin phenol reagent. 193 265–275. 14907713

[B33] Lucena G. M. Franco J. L. Ribas C. M. Azevedo M. S. Meotti F. C. Gadotti V. M. (2007). *Cipura paludosa* extract prevents methyl mercury-induced neurotoxicity in mice. 101 127–131. 10.1111/j.1742-7843.2007.00091.x 17651315

[B34] Maodaa S. N. Allam A. A. Ajarem J. Abdel-Maksoud M. A. Al-Basher G. I. Wang Z. Y. (2016). Effect of parsley (*Petroselinum crispum*, Apiaceae) juice against cadmium neurotoxicity in albino mice (*Mus musculus*). 12:6. 10.1186/s12993-016-0090-3 26846273 PMC4743362

[B35] Mello-Carpes P. B. Barros W. Borges S. Alves N. Rizzetti D. Peçanha F. M. (2013). Chronic exposure to low mercury chloride concentration induces object recognition and aversive memories deficits in rats. 31 468–472. 10.1016/j.ijdevneu.2013.05.009 23770019

[B36] Ministry of the Environment from Japan (2013). Chiyoda: Ministry of the Environment from Japan 1–64.

[B37] Moneim A. A. E. (2015). The neuroprotective effect of berberine in mercury-induced neurotoxicity in rats. 30 935–942. 10.1007/s11011-015-9652-6 25600690

[B38] National Research Council of the National Academies (2011). 8th Edn. Washington, DC: The National Academy Press 1–246.

[B39] Oliveira A. C. Pereira M. C. Santana L. N. Fernandes R. M. Teixeira F. B. Oliveira G. B. (2015). Chronic ethanol exposure during adolescence through early adulthood in female rats induces emotional and memory deficits associated with morphological and molecular alterations in hippocampus. 29 712–724. 10.1177/0269881115581960 25922423

[B40] Oliveira G. B. Fontes E. A. Jr. de Carvalho S. da Silva J. B. Fernandes L. M. Oliveira M. C. (2014). Minocycline mitigates motor impairments and cortical neuronal loss induced by focal ischemia in rats chronically exposed to ethanol during adolescence. 1561 23–34. 10.1016/j.brainres.2014.03.005 24637259

[B41] Omanwar S. Ravi K. Fahim M. (2011). Persistence of EDHF pathway and impairment of the nitric oxide pathway after chronic mercury chloride exposure in rats: mechanisms of endothelial dysfunction. 30 1777–1784. 10.1177/0960327110391389 21148200

[B42] Omanwar S. Saidullah B. Ravi K. Fahim M. (2013). Vasorelaxant effects of mercury on rat thoracic aorta: the nitric oxide signaling mechanism. 33 904–910. 10.1177/0960327113512341 24347300

[B43] Ostertag S. K. Stern G. A. Wang F. Lemes M. Chan H. M. (2013). Mercury distribution and speciation in different brain regions of beluga whales (*Delphinapterus leucas*). 456-457 278–286. 10.1016/j.scitotenv.2013.03.106 23624002

[B44] Peixoto N. C. Pereira M. E. (2007). Effectiveness of ZnCl2 in protecting against nephrotoxicity induced by HgCl2 in newborn rats. 66 441–446. 10.1016/j.ecoenv.2006.02.012 16620979

[B45] Rice K. M. Walker E. M. Jr. Wu M. Gillette C. Blough E. R. (2014). Environmental mercury and its toxic effects. 47 74–83. 10.3961/jpmph.2014.47.2.74 PMC3988285 24744824

[B46] Sanes J. N. Donoghue J. P. (2000). Plasticity and primary motor cortex. 23 393–415. 10.1146/annurev.neuro.23.1.393 10845069

[B47] Skerfvingi S. B. Copplestone J. F. (1976). Poisoning caused by the consumption of organomercury-dressed seed in Iraq. 54 101–112. 1087584 PMC2366450

[B48] Suzuki T. Akagi H. Akimura K. Ando T. Sakamoto M. Satoh H. (2004). Brasília: Ministério do Meio Ambiente 1–119.

[B49] Szász A. Barna B. Gajda Z. Galbác G. Kirsch-Volders M. Szente M. (2002). Effects of continuous low-dose exposure to organic and inorganic mercury during development on epileptogenicity in rats. 23 197–206. 10.1016/S0161-813X(02)00022-0 12224761

[B50] Szumañska G. Gadamski R. Albrecht J. (1993). Changes of the Na/K ATPase activity in the cerebral cortical microvessels of rat after single intraperitoneal administration of mercuric chloride: histochemical demonstration with light and electron microscopy. 86 65–70. 10.1007/BF00454900 8396838

[B51] Teixeira F. B. Fernandes R. M. Farias-Junior P. M. Costa N. M. Fernandes L. M. Santana L. N. (2014a). Evaluation of the effects of chronic intoxication with inorganic mercury on memory and motor control in rats. 11 9171–9185. 10.3390/ijerph110909171 25198682 PMC4199013

[B52] Teixeira F. B. Santana L. N. Bezerra F. R. De Carvalho S. Fontes-Júnior E. A. Prediger R. D. (2014b). Chronic ethanol exposure during adolescence in rats induces motor impairments and cerebral cortex damage associated with oxidative stress. 9:e101074. 10.1371/journal.pone.0101074 24967633 PMC4072717

[B53] Trebucobich M. S. Hazelhoff M. H. Chevalier A. A. Passamonti S. Brandoni A. M. Torres A. M. (2014). Protein expression of kidney and liver bilitranslocase in rats exposed to mercuric chloride–a potential tissular biomarker of toxicity. 225 305–310. 10.1016/j.toxlet.2013.11.022 24374050

[B54] Triunfante P. Soares M. E. Santos A. Carmo S. T. H. Bastos M. L. (2009). Mercury fatal intoxication: two case reports. 184 e1–e6. 10.1016/j.forsciint.2008.10.023 19070443

[B55] Wang S. Lv Q. Yang Y. Guo L. H. Wan B. Ren X. (2016). Arginine decarboxylase: a novel biological target of mercury compounds identified in PC12 cells. 118 109–120. 10.1016/j.bcp.2016.08.019 27565891

